# Haptic-Motor Transformations for the Control of Finger Position

**DOI:** 10.1371/journal.pone.0066140

**Published:** 2013-06-06

**Authors:** Daisuke Shibata, Jason Y. Choi, Juan C. Laitano, Marco Santello

**Affiliations:** 1 Kinesiology Program, College of Health Solutions, Arizona State University, Tempe, Arizona, United States of America; 2 School of Biological and Health Systems Engineering, Arizona State University, Tempe, Arizona, United States of America; University of Reading, United Kingdom

## Abstract

Dexterous manipulation relies on modulation of digit forces as a function of digit placement. However, little is known about the sense of position of the vertical distance between finger pads relative to each other. We quantified subjects' ability to match perceived vertical distance between the thumb and index finger pads (*d_y_*) of the right hand (“reference” hand) using the same or opposite hand (“test” hand) after a 10-second delay without vision of the hands. The reference hand digits were passively placed non-collinearly so that the thumb was higher or lower than the index finger (*d_y_* = 30 or –30 mm, respectively) or collinearly (*d_y_* = 0 mm). Subjects reproduced reference hand *d_y_* by using a congruent or inverse test hand posture while exerting negligible digit forces onto a handle. We hypothesized that matching error (reference hand *d_y_* minus test hand *d_y_*) would be greater (a) for collinear than non-collinear *d_y_*s, (b) when reference and test hand postures were not congruent, and (c) when subjects reproduced *d_y_* using the opposite hand. Our results confirmed our hypotheses. Under-estimation errors were produced when the postures of reference and test hand were not congruent, and when test hand was the opposite hand. These findings indicate that perceived finger pad distance is reproduced less accurately (1) with the opposite than the same hand and (2) when higher-level processing of the somatosensory feedback is required for non-congruent hand postures. We propose that erroneous sensing of finger pad distance, if not compensated for during contact and onset of manipulation, might lead to manipulation performance errors as digit forces have to be modulated to perceived digit placement.

## Introduction

Dexterous manipulation relies on the ability to coordinate digit forces [1], [2] and positions [3]–[8]. Choice of digit placement plays an important role in manipulation, as indicated by its sensitivity to task, object geometry, and intended manipulation [3], [6], [8]–[11]. It has recently been shown that when subjects are asked to manipulate objects that do not constrain digit placement at specific locations, trial-to-trial variability in digit placement is compensated by concurrent modulation of digit forces such that manipulation can be performed in a consistent fashion [6]. These findings indicate that the central nervous system integrates the sense of digit position with motor commands responsible for distributing forces among the digits [2], [12], [13].

Although it could be argued that vision of hand placement on the object would play a key role in the modulation of digit forces as a function of position, the position of one or more digits is often occluded by the object as it happens when grasping a bottle or holding a cup. However, a recent study has shown that removal of visual feedback of thumb position before object contact does not significantly affect thumb placement relative to the index finger [14]. Furthermore, psychophysical evidence from matching finger span to visually or haptically perceived object size indicates that the horizontal distance between the finger pads can be accurately sensed without visual feedback of the hand in the absence of contact forces [15]. Similarly, the horizontal distance between the thumb and two fingers was accurately matched even when the matching task was performed with the contralateral hand while holding an object so as to prevent it from slipping without visual feedback of both hands and the object [16]. These observations suggest that visuomotor transformations mapping object graspable surfaces to relative fingertip position or grip axis orientation can be accurately implemented using only somatosensory feedback.

The above studies, however, constrained grasp aperture to change only on one axis (horizontal) [15], [16] or focused on the orientation of contacts on the horizontal plane [14]. Therefore, the extent to which the above findings apply to tasks involving *non-collinear* contacts, eliciting different patterns of mechanoreceptor activity than collinear contacts, remains to be established. Non-collinear contacts occur when normal forces exerted by opposing digits are used to generate a torque while grasping an object. This is achieved by an offset between the contact points in the plane of the contact surfaces. This is an important question because object manipulation often does not constrain the finger pads to be positioned collinearly relative to each other [5], [6]. Another gap in previous literature is that digit force was not measured, hence not controlled for, by studies that allowed contact forces [14], [16], [17]. Therefore, it is not known whether subjects' ability to accurately reproduce digit contact orientation without visual feedback might have been associated with exerting specific force magnitudes.

Another open question is whether the ability to reproduce digit position depends on whether sensing occurs through the same versus the opposite hand. Lastly, although the effects of non-congruent arm position on perception of hand shape using the opposite hand were previously addressed [18], it is not known whether congruence of relative position of the digits affects subjects' ability to match finger pad distance haptically perceived with the opposite hand. It should be emphasized that the haptic-motor transformations associated with reproducing finger pad distance rely on different abilities depending on whether the posture of the hand used for sensing finger pad distance is the same or different from the posture of the hand used for matching. Specifically, when the posture of the ‘sensing’ and ‘matching’ hand are the same, subjects can use the memory of somatosensory feedback acquired at a given posture to reproduce the same posture of the ‘matching’ hand, hence finger pad distance. In contrast, when the postures of the ‘sensing’ and ‘matching’ hands differ, somatosensory feedback arising from muscles, tendons, and skin afferents needs to be processed to create an appropriate internal representation of the relative position of the finger pads independent from postural sensory cues.

**Figure 1:**
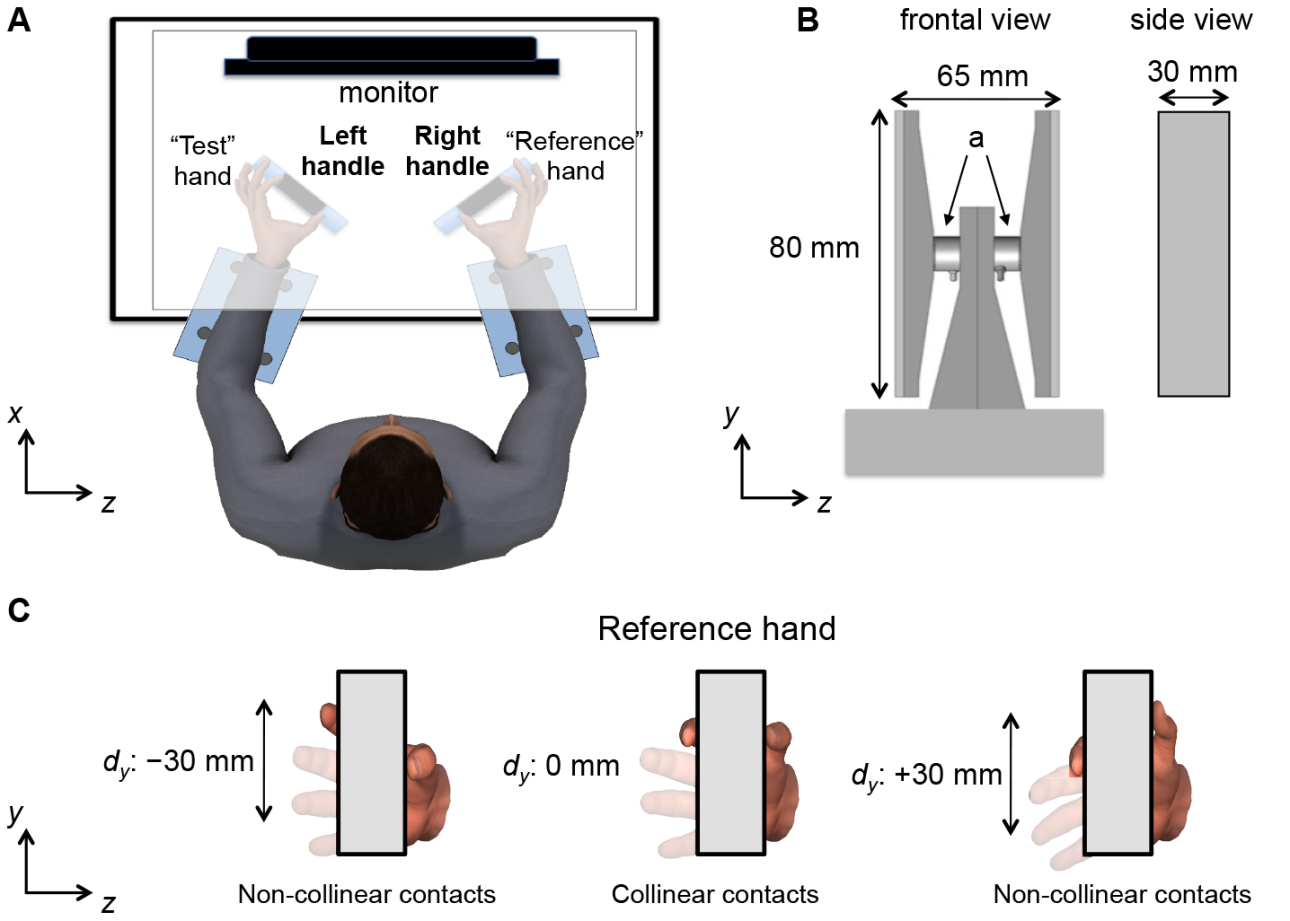
Experimental setup. Panel A shows a top view of the experimental setup. In this figure, the subject is shown performing the matching task using the left hand (“Test” hand) to reproduce the vertical distance (*d_y_*) between the thumb and index finger pad of the right hand (“Reference” hand) (see text for more details). Note that the table top (gray) prevented the subjects from seeing their forearms and hands but is shown as transparent for graphical purposes only. Forearms and wrists were strapped to the table to prevent movements within and across trials while the handles were anchored to the table. Panel B shows a frontal view of one of the two handles used for the study (“*a*” denotes force/torque sensors). Panel C shows the frontal view of the handle with the three *d_y_*s of the reference hand used for the study. Note that *d_y_* is defined as positive or negative when the thumb pad is higher or lower than the index finger pad, respectively.

**Figure 2:**
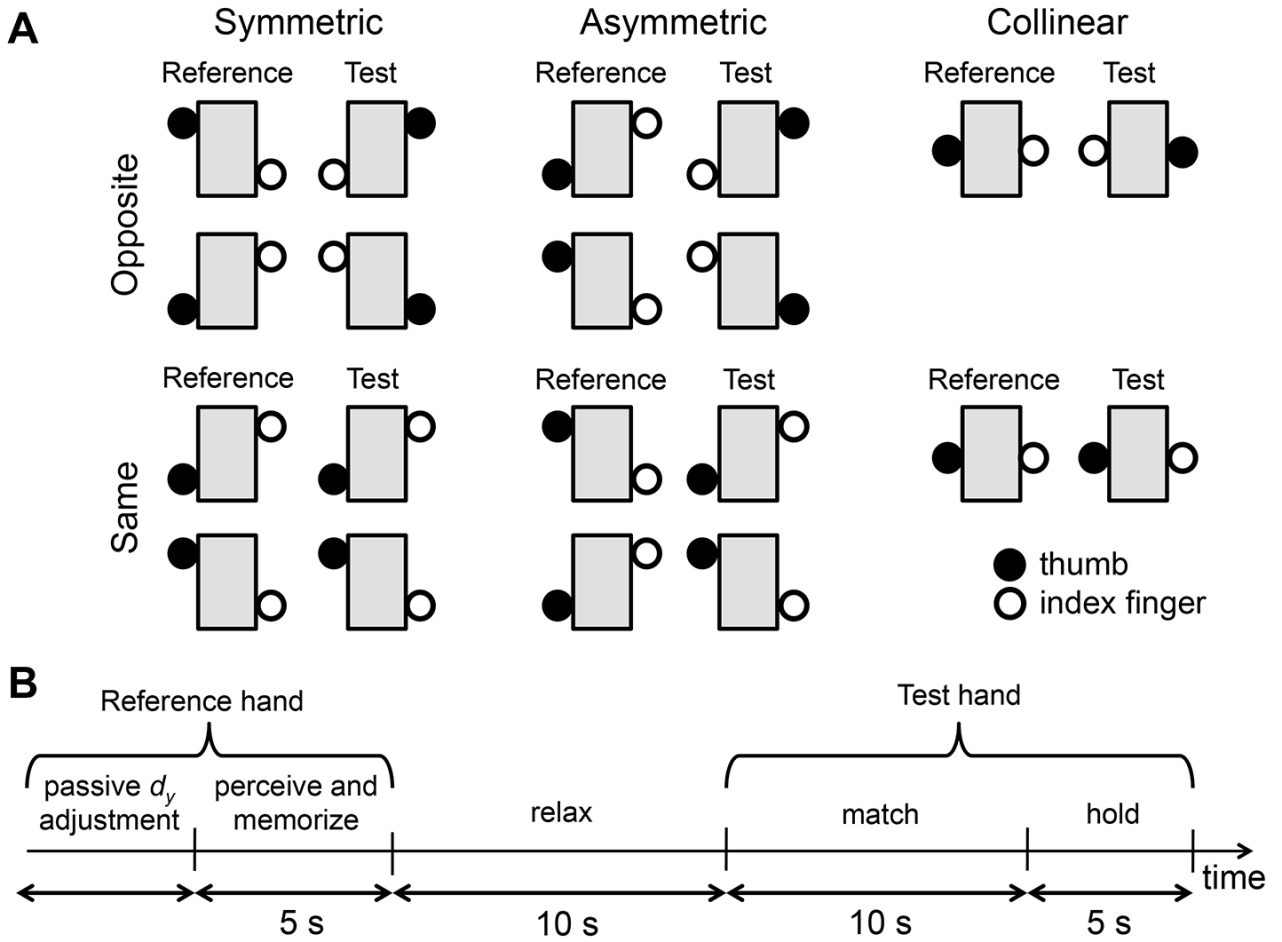
Experimental conditions and trial timeline. Panel A shows all experimental conditions. The thumb and index finger of the reference hand were positioned at one of the three target vertical distances (*d_y_*; see Figure 1) and subjects were asked to reproduce *d_y_* after a 10-second delay using either the opposite hand (test hand) (“Opposite” condition) or the same hand (reference hand) (“Same” condition). For both Opposite and conditions, subjects were asked to either reproduce *d_y_* using the congruent reference hand posture (“Symmetric” condition) or an inverse posture (“Asymmetric” condition) (see text for more details). Note that the collinear *d_y_* requires subjects to use the same posture with both hands. Panel B shows the trial timeline. In the phase of “passive *d_y_* adjustment”, the digits were passively placed to one of three digit positions. Once the desired *d_y_* was reached and digit force was controlled, recoding of reference hand *d_y_* started while subjects tried to perceive and memorize the reference hand *d_y_* for 5 seconds. During the “relax” phase, subjects were asked to retain the remembered *d_y_* while relaxing their hands for 10 seconds, followed by the “match” phase in which they had to reproduce that *d_y_* with test hand within 10 seconds. The test hand *d_y_* was then recorded for 5 seconds while subjects kept the digit position and digit force level (“hold” phase).

The present study was designed to address the above gaps by determining the factors that affect subjects' ability to sense and reproduce the vertical distance between finger pads. Specifically, we asked subjects to sense the vertical distance between the center of pressure (CoP) of the thumb and index finger pads (*d_y_*) of the right hand (“reference” hand) and, after a brief delay, match it using the same or opposite hand (“test” hand). In addition, we asked subjects to perform the matching task using an inverse test hand posture relative to the reference hand to prevent them from merely matching hand postures (Fig. 1C and 2A). An inverse hand posture is generated by changing the relative vertical position of the two digits without involving wrist supination or pronation.

**Figure 3:**
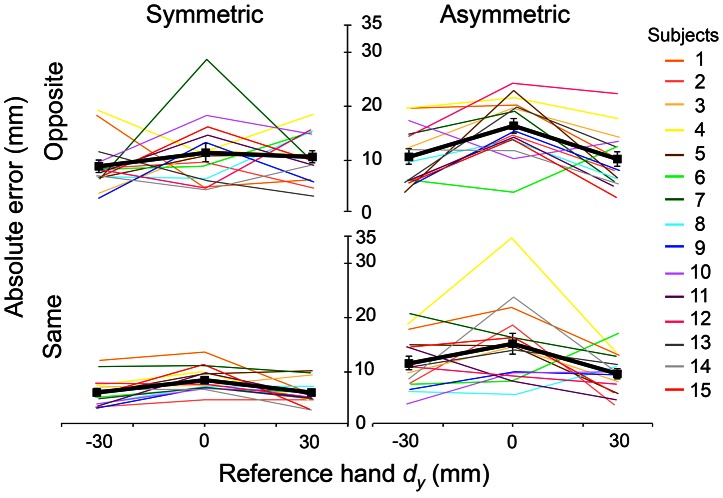
Absolute error: individual subjects. Averages of absolute error of 5 trials from all subjects are shown as a function of reference hand *d_y_* (+30, 0, and −30 mm) for the four matching conditions, and connected with different colors for each subject. The thick black line denotes the mean absolute error averaged across 15 subjects with standard error of the mean. Top panels show the opposite condition, in which subjects were asked to reproduce *d_y_* using the opposite hand after a brief delay. Bottom panels show the same condition, in which subjects used the same hand to reproduce *d_y_* after a brief delay. These two conditions are shown separately for the symmetric (left) and asymmetric (right) condition, in which postures of the reference and test hand were congruent and inverse, respectively.

We hypothesized that the matching error (difference between reference and test hand *d_y_*) would be greater *(1)* in the collinear (*d_y_* = 0 mm) than non-collinear (*d_y_*≠0 mm) digit position (Fig. 1C), *(2)* when the postures of the reference and test hand were inversed (Asymmetric, middle column, Fig. 2A), and *(3)* when subjects reproduced finger pad distance using the opposite hand (top row, Fig. 2A) as opposed to using the same hand (bottom row, Fig. 2A). The rationale for the first hypothesis is that somatosensory afferent responses from skin, joints, muscles, and tendons would provide signals with higher signal-to-noise ratio about finger pad distance when finger pads are further apart than when they are collinear [19]–[22]. The second hypothesis is based on the expectation that matching finger pad distance would be facilitated by matching (remembered) sensory feedback from reference hand to sensory feedback from test hand when hand postures are congruent. Therefore, this hypothesis also implies that subjects' ability to match finger pad distance would be challenged by perceiving and reproducing finger pad distance dissociated from hand postural cues, i.e., reproducing a posture-independent internal representation of finger pad distance, for incongruent hand postures (Symmetric vs. Asymmetric, Fig. 2A). The rationale for the third hypothesis is that transferring sensory information across cerebral hemispheres to generate motor commands with the opposite hand would add sensorimotor transformation errors relative to those associated to perceiving and reproducing finger pad distance with the same hand [23], [24].

## Materials and Methods

### Subjects

Fifteen right-handed volunteers (10 males and 5 females, mean age and standard deviation: 23.5±4.5 yrs) participated in this study. Hand dominance was assessed using the 10-item Edinburgh Handedness Inventory [25]. All subjects were classified as right-handed (mean Laterality Quotient and standard deviation: 83.3±22.3). Subjects were naïve to the purpose of the study and had no previous history of orthopedic, neurological trauma, or pathology of the upper limbs. Subjects gave their written informed consent according to the declaration of Helsinki and the protocols were approved by the Office of Research Integrity and Assurance at Arizona State University.

### Apparatus

Subjects sat on an adjustable chair with both forearms resting on a table. A tabletop, in which a computer monitor was placed at subjects' eye level, was used to prevent vision of the forearms, hands, and the two identical handles used to measure digit forces and torques exerted by thumb and index finger (Fig. 1A; see below for details). After matching the position and orientation of the arms and hands, the forearms and wrists were constrained with straps and rigid dowels anchored to the platform to minimize movements across trials and throughout the experiment (Fig. 1A). The relation between the hand posture and the handle position was also maintained constant by anchoring the handles to the table. The positioning of the object and platform was adjusted for each subject and fixed after we confirmed that subjects' digits were placed on the handle in a comfortable posture. The CoP of the thumb pad and index finger pad of each hand was computed as described in Fu et al. (2010) using two six-component force/torque sensors mounted on each side of both handles (ATI Nano-25 SI-125-3, ATI Industrial Automation, Garner, NC; force range: 125, 125, and 500 N for *x-*, *y-* and *z-*axes, respectively; force resolution: 0.06 N; torque range: 3000 N•mm; torque resolution: 0.378 N•mm; “a”, Fig. 1B). The CoP was defined as the vertical coordinates of the center of pressure of the contact between the finger pad and the graspable surface (Fig. 1B) relative to the center of the sensor. Calibration of each sensor with its contact surface revealed that the vertical (*y*) coordinate of each digit CoP could be computed with a maximum error across all measurements and sensors of±1.1 mm (maximum average error±standard deviation: 0.2±0.5 mm) when three forces (0.6, 1.0, and 1.4 N) were applied perpendicular to the contact surface mounted on the sensor. The actual normal force that subjects exerted with a digit during the experimental tasks fell within the 0.6–1.4 N in 95% of all trials. Error in CoP reconstruction was similar across the four sensors. The contact surfaces of the handles were covered with 100-grit sandpaper (static friction coefficient range: 1.4–1.5) to allow subjects to maintain a relaxed posture of the digits without having to exert significant forces on the handles to prevent the digits from slipping. As a result, tangential forces were very small and ranged between 0.1 and 0.2 N. Force and torque data were acquired, recorded, and stored in a computer with a 12-bit A/D converter board (PCI-6225, National Instruments, Austin, TX; sampling frequency: 1 kHz) through a custom data acquisition interface (LabVIEW version 8.0, National Instruments).

### Experimental Procedures

We asked subjects to match the vertical distance (*d_y_*) between thumb and index CoP of the right (dominant) hand (“reference” hand) using the same hand or the opposite hand (both are defined as “test” hand). At the beginning of the experiment, subjects performed several practice trials to familiarize themselves with the task. Note that feedback about matching performance was not provided during the practice or experimental trials.

#### Reference Hand

We tested three *d_y_*s at the reference hand: +30, 0, and −30 mm, defined as higher, same, or lower thumb CoP relative to index finger CoP (Fig. 1C). During the practice trials, we confirmed that all subjects could comfortably achieve these non-collinear *d_y_*s (+30 and −30 mm) within their range of motion regardless of variability of hand size. We measured three parameters of reference hand: (1) length, defined as the distance from the wrist crease to the tip of middle finger (average length±standard deviation: 184.2±10.6 mm); (2) width, defined as the distance between the radial aspect of the second metacarpo-phalangeal (*mcp*) joint and the ulnar aspect of the fifth *mcp* joint (average width±standard deviation: 83.1±4.8 mm); and (3) thumb-index distance, defined as the distance between outstretched thumb and index fingertips (average length±standard deviation: 163±13.1 mm). No outliers were found for any of these three parameters across subjects.

The experimenter asked subjects to relax the digits of the reference hand while passively moving them to one of the three *d_y_*s (“passive *d_y_* adjustment”, Fig. 2B). During this procedure and while matching *d_y_* (see Fig. 1C), subjects were required to keep the middle, ring, and little fingers extended. One of the experimenters monitored the CoP for each digit and the resultant *d_y_* of the reference hand on a second computer monitor that was not visible to the subject. Another experimenter visually verified that subjects maintained the desired hand posture (thumb and index fingertips in contact with the device while keeping the other fingers extended) until the desired *d_y_* was reached. While keeping a given *d_y_*, we asked subjects to generate very small normal forces with the thumb and index finger of reference hand. This criterion was enforced by providing visual feedback of digit normal forces to the subject on a computer monitor placed on the tabletop (Fig. 1A). The normal force range was between 0.4 and 1 N, the lower bound being the minimum force required for accurate computation of digit CoP [6]. Once this force criterion was met, we asked subjects to maintain reference hand *d_y_* for 5 seconds within a tolerance window of±5 mm from the desired *d_y_* in order to start recording reference hand *d_y_* (“perceive and memorize”, Fig. 2B). Throughout the experiment, subjects were able to maintain each of the three prescribed *d_y_*s within the±5 mm tolerance window. After the 5-seconds period, we gave subjects a verbal signal to release the digits of reference hand from the handle and place the hand flat (all digits straight, adducted, and with the palm in a horizontal orientation) on the table. Note that neither hand was in contact with the handle for 10 seconds (“relax”, Fig. 2B). After the 10 seconds delay, we gave another verbal signal to match the remembered reference hand *d_y_* using test hand within 10 seconds (see below for details). Note also that, when one hand was in contact with the handle, the other hand was placed flat on the table.

#### Test Hand

Subjects were asked to actively place test hand to the remembered *d_y_* on its respective handle after the verbal signal was given within 10 seconds (“match”, Fig. 2B). During the “match” period, subjects were required to exert normal forces between 0.4 and 1 N (see above). The trial was repeated if subjects were unable to exert digit forces within the required target during the “match” period. When the force criterion was met within the 10-second period, subjects were given a verbal signal to hold *d_y_* for 5 seconds to record the test hand thumb and index finger CoP (“hold”, Fig. 2B). Finally, subjects were asked to release the test hand from the handle after another verbal signal was given.

We tested four matching conditions that differed depending on whether test hand and reference hand were required to assume a congruent or inverse posture (“Symmetric” and “Asymmetric” conditions, respectively) and whether matching tasks were to be performed with the same hand used as the reference hand or the opposite hand (“Same” and “Opposite” conditions, respectively). For each of these four conditions, we tested the above-described three *d_y_*s (Fig. 1C).

In the Symmetric condition (Fig. 2A, left column), subjects matched the reference hand *d_y_* with the test hand by keeping the relative digit position congruent across the two hands. Specifically, when subjects detected the thumb CoP to be higher or lower than the index finger CoP of the reference hand, they were asked to position the thumb CoP higher or lower than the index finger CoP of the test hand, respectively, while matching the reference hand *d_y_*. For the Asymmetric condition (Fig. 2A, middle column), subjects were asked to match reference hand *d_y_* by using an inverse relative digit position with the test hand. Specifically, when subjects detected the thumb CoP to be higher or lower than the index CoP of the reference hand, they were asked to position the thumb CoP lower or higher than the index CoP of the test hand, respectively. Note that for the collinear digit position (*d_y_* = 0), the test hand *d_y_* (Fig. 2A, right column) reflects the perceived reference hand *d_y_*. Therefore, even though the actual reference hand *d_y_* is∼0, they might have perceived *d_y_* to be non-zero. If so, subjects would reproduce *d_y_* with test hand by positioning thumb and index finger CoP in a non-collinear configuration that might be symmetrical or asymmetrical depending on the perceived relative position of reference hand *d_y_*.

Subjects were notified whether the postures of test hand and reference hand were required to be congruent or inverse and whether the test hand was the opposite or same hand before starting the block of consecutive trials. Each block of the four experimental conditions consisted of 15 consecutive trials (5 trials per *d_y_*; Fig. 1C) for a total of 60 trials. For each experimental condition, the order of presentation of reference hand *d_y_* was randomized across trials and subjects. The presentation of experimental conditions was counterbalanced across subjects.

### Data Processing

Force data were filtered using a moving average filter every 50 samples over the duration of data recording and used for computing and displaying online normal force magnitude and digit CoPs and *d_y_* for both reference and test hand using LabVIEW. The CoP of each digit was defined as the vertical coordinate of the CoP of the contact between the finger pad (thumb or index finger) and the surface of the handle relative to the center of the force/torque sensor (Fig. 1B). After data collection, CoP data for each digit were analyzed off-line with custom-written software (Matlab, The MathWorks, Natick, MA). The vertical coordinate of digit CoP was averaged within each trial for each digit and was used to compute *d_y_* for statistical analysis.

Error in matching performance was defined as *d_y_* of test hand during the “hold” phase minus reference hand *d_y_* during the “perceive and memorize” phase (Fig. 2B) and was computed as either absolute or relative error. The relative error takes into consideration the sign of *d_y_* of the reference and test hand, and therefore can take a positive or negative value. The sign of the relative error denotes whether subject made over- or under-estimation of the reference hand *d_y_* in the non-collinear conditions. In contrast, absolute error was computed by taking the absolute value of positive and negative relative errors. Over- and under-estimation of reference hand *d_y_* were defined as longer and shorter distances, respectively, between the thumb and index finger CoP of test hand relative to that of the reference hand. The sign of the relative error for non-collinear *d_y_* depends on the sign convention used for reference hand *d_y_*. Specifically, when reference hand thumb was passively placed non-collinearly and higher than the index finger (*d_y_*≈30 mm), negative and positive relative error indicate under- and over-estimation of reference hand *d_y_*, respectively. In contrast, when reference hand thumb was placed lower than the index finger (*d_y_*≈–30 mm), negative and positive relative error indicate over- and under-estimation of reference hand *d_y_*, respectively. Analysis of relative error in the collinear reference hand *d_y_*s was excluded from analysis because, unlike the non-collinear reference hand *d_y_*s (above), reference hand *d_y_* could fluctuate between positive and negative values across trials.

### Statistical Analysis

After data processing for the computation of absolute and relative error, we determined whether there were outliers within each subject and experimental condition. Outliers were defined as data above or below three standard deviations of the mean. We found only one outlier datum and excluded it from statistical analysis. Statistical analysis with and without the outlier datum did not change the statistical main effects and interactions.

To determine the extent to which actual reference hand *d_y_* could be grouped within each of the three desired *d_y_*s for statistical analyses, we performed linear regression analysis on reference hand *d_y_* versus test hand *d_y_* on separate group of trials (*n* = 5) from each desired *d_y_*, experimental condition, and subject. This analysis was performed to determine the extent to which trial-to-trial deviations from the desired reference hand *d_y_* within the±5 mm tolerance window were large enough to be perceived by the subject as detectable by systematic changes in test hand *d_y_*. Furthermore, to determine whether trial-to-trial fluctuation of reference hand force induced systematic changes in the test hand force and matching error, linear regression analyses were also performed on the reference hand force versus the test hand force across subjects (*n* = 15) and matching error within subjects (*n* = 60). We also performed linear regression analysis on the absolute error over 60 trials within subjects to determine whether subjects' ability to match the digit positions varied systematically throughout the duration of the experiment.

E*_abs_* was analyzed using 3-way analysis of variance (ANOVA) with repeated measures within *d_y_* (3 levels: +30, 0, −30 mm), test hand posture (2 levels: Symmetric, Asymmetric), and Hand (2 levels: Opposite, Same). These within-subject factors were used to test the effect of each experimental condition on *d_y_* matching accuracy. This 3-way ANOVA was performed at the *p*≤0.05 significance level to test the hypotheses that the matching error would be greater *(1)* for collinear than non-collinear digit positions, *(2)* when the postures (relative positions of thumb and index finger) of the reference and test hand were inversed, and *(3)* when subjects reproduced finger pad distance using the opposite hand as opposed to using the same hand. A *post hoc* test was used to test the hypothesis that the matching error would be greater in the collinear (*d_y_* = 0 mm) than non-collinear (*d_y_*≠0 mm) digit position. *Post hoc* tests were run using paired sample *t*-tests with Bonferroni corrections when appropriate. Relative error from non-collinear *d_y_*s was analyzed by two-tailed *t*-tests for each experimental condition and non-collinear reference hand *d_y_*s to determine whether the mean relative error was significantly different from zero.

Sphericity assumptions were tested for all analyses of absolute and relative error (Greenhouse-Geisser analysis). Violations of normality equality assumptions were tested using Shapiro-Wilk test and Levene's test, respectively (*p*>0.05). Values in the text are reported as means±standard error.

## Results

### Validation of Experimental Protocol

#### Effect of Small Trial-to-trial Fluctuations in Reference Hand dy

Linear regression analysis on reference hand *d_y_* versus test hand *d_y_* revealed that virtually all linear fits (>95%) were not statistically significant (*p*>0.05). Therefore, as the small trial-to-trial fluctuations in reference hand *d_y_* did not elicit systematic changes in test hand *d_y_*, for statistical purposes we allocated measured reference hand *d_y_* values to its corresponding category (0, +30 mm, or −30 mm).

#### Effect of Small Trial-to-trial Fluctuations in Reference Hand Force

The average normal forces of the thumb and index finger exerted by reference and test hand were virtually identical (0.78±0.05 N and 0.80±0.05 N, respectively). The linear regression analysis revealed that the reference and test hand normal forces were highly correlated (*r^2^* = 0.92, *p*<0.01). Linear regression analysis on the reference hand normal force versus matching error within subjects revealed that linear fits from 14 out of 15 subjects were not statistically significant (*p*>0.05). For the only subject for whom the linear fit was statistically significant (*p*<0.05) the *r^2^* value was only 0.11. Thus, these two linear regression analyses indicate that there was no systematic change in the test hand force or matching error as a function of the small trial-to-trial fluctuations of reference hand normal force.

#### Effect of Experiment Duration

The linear regression analysis on the matching error over 60 trials within subjects revealed that 10 out of 15 (66.6%) linear fits were not statistically significant (*p*>0.05). The remaining 5 out 15(33.4%) linear fits that were statistically significant (*p*<0.05) were characterized by an inconsistent sign of the regression coefficients. Most importantly, 13 out of 15 (86.7%) of the *r^2^* of the significant linear fits was very small (<0.1), whereas the maximum *r^2^* of the remaining 2 out of 15 (13.3%) significant fits was only 0.13. Therefore, matching error did not systematically vary as a function of trial. Thus, we could rule out effects of the duration of experiment, such as fatigue or familiarization with task, on matching error.

### Absolute Error

Figure 3 shows the averages of absolute matching error of 5 trials from all subjects as a function of the vertical distance between thumb and index finger CoP (*d_y_*) for each experimental condition. The matching errors per vertical distance were connected using different colors for each subject, and the thick black line denotes the mean absolute error averaged across 15 subjects. Overall, subjects tended to make greater absolute error when asked to match collinear *d_y_* (*d_y_* = 0 mm) than when the thumb CoP was placed higher (*d_y_* = 30 mm) or lower (*d_y_* = −30 mm) than the index finger CoP. Furthermore, greater absolute error were produced when the postures of the test and reference hand were inversed (Asymmetric condition) and when the matching task was performed with the opposite hand (Opposite condition). The performance of two subjects (#7 and #4) was characterized by large errors for collinear *d_y_* in Opposite-Symmetric and Same-Asymmetric conditions (dark green and yellow lines, respectively, in Fig. 3). Thus, we performed the statistical analyses both with and without these two subjects. The statistical main effects were not altered by removing these two subjects, thus all statistical analyses reported below were performed on all subjects.

**Figure 4:**
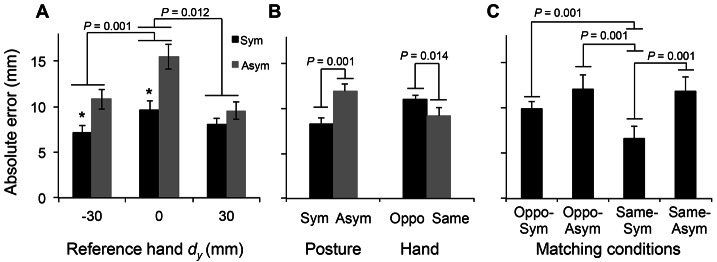
Absolute error: average data. Absolute errors were compared across reference hand *d_y_*s, postures, hands, and matching conditions. Panel A shows average absolute error for symmetric and asymmetric conditions (black and gray bars, respectively) across reference hand *d_y_*s. Panel B, left, shows average absolute error for symmetric and asymmetric conditions (black and gray bars, respectively) as a function of hand posture (Sym, Asym: Symmetric and Asymmetric conditions, respectively). Panel B, right, shows average absolute error when reference and test hand differed or were the same (Oppo, Same: Opposite and Same conditions, respectively). Panel C shows absolute error averaged for each condition. For all panels, absolute errors were averaged across all subjects within the given comparisons groups (±SE). The asterisks denote significant difference (*p*<0.05) between the symmetric and asymmetric conditions.

Greater absolute error in the collinear than non-collinear *d_y_* was observed in both symmetric and asymmetric matching conditions (black and gray bars, Fig. 4A). Three-way ANOVA confirmed that absolute error was significantly greater in the collinear than non-collinear conditions (12.6±0.9 mm for *d_y_* = 0 mm; 9.0±0.9 mm for *d_y_* = −30 mm; 8.8±0.7 for *d_y_* = 30 mm; main effect of Distance: F[_2,28_] = 10.8; *p*<0.01; Fig. 4A), and in the asymmetric than symmetric condition (12.0 ±0.9 mm and 8.3±0.5 mm, respectively; main effect of Posture: F[_1,14_] = 26.5; *p*<0.01; Fig. 4B, left). We also found a significant interaction Posture × Distance (7.1±0.8 mm for Symmetric at *d_y_* = −30 mm; 9.6±1.0 mm for Symmetric at *d_y_* = 0 mm; 8.1±0.7 mm for Symmetric at *d_y_* = 30 mm; 10.8±1.1 mm for Asymmetric at *d_y_* = −30 mm; 15.5±1.4 mm for Asymmetric at *d_y_* = 0 mm; 9.6±1.0 mm for Asymmetric at *d_y_* = 30 mm; F[_2,28_] = 4.02; *p*<0.05; Fig. 4A).

Absolute error was significantly greater in the Asymmetric than Symmetric condition for *d_y_* = 0 and −30 mm (*post hoc t-*test: Symmetric at *d_y_* = 0 mm vs. Asymmetric at *d_y_* = 0 mm; t[_14_] = −3.54, *p*<0.003; Symmetric at *d_y_* = −30 mm vs. Asymmetric at *d_y_* = −30 mm; t[_14_] = −6.17; *p*<0.001; adjusted α = 0.003; Fig. 4A). This indicates that the main effect of Posture (Fig. 4B, left) arose from the difference in the absolute error between the symmetric and asymmetric conditions during the *d_y_* = 0 and −30 mm, but not 30 mm. Moreover, greater absolute error were found when matching was performed by the opposite hand than by the same hand (11.0±0.8 mm and 9.2±0.7 mm respectively; main effect of Hand: F[_1,14_] = 7.907; *p*<0.05; Fig. 4B, right).

We also found a significant interaction Hand × Posture (10.0±0.7 mm for Opposite-Symmetric; 12.1±1.6 mm for Opposite-Asymmetric; 6.6±1.4 mm for Same-Symmetric; and 11.8±1.6 mm for Same-Asymmetric; F[_1,14_] = 5.411; *p*<0.05; Fig. 4C). *Post hoc* paired *t-*tests with Bonferroni corrections found that subjects made significantly smaller E*_abs_* when matching was performed by the same hand in the symmetric condition (Same-Symmetric) than the Opposite-Symmetric, Opposite-Asymmetric and Same-Asymmetric conditions (t[_14_] = −4.808, −5.724, and −5.878, respectively; *p*<0.001 for all conditions; adjusted α = 0.008; Fig. 4C). Note that no significant difference was found for pairwise comparisons across the other three experimental conditions. This indicates that subjects' ability to match reference hand *d_y_* was greatest when sensing and matching was performed with the same hand and using the same hand posture.

### Relative Error

**Figure 5:**
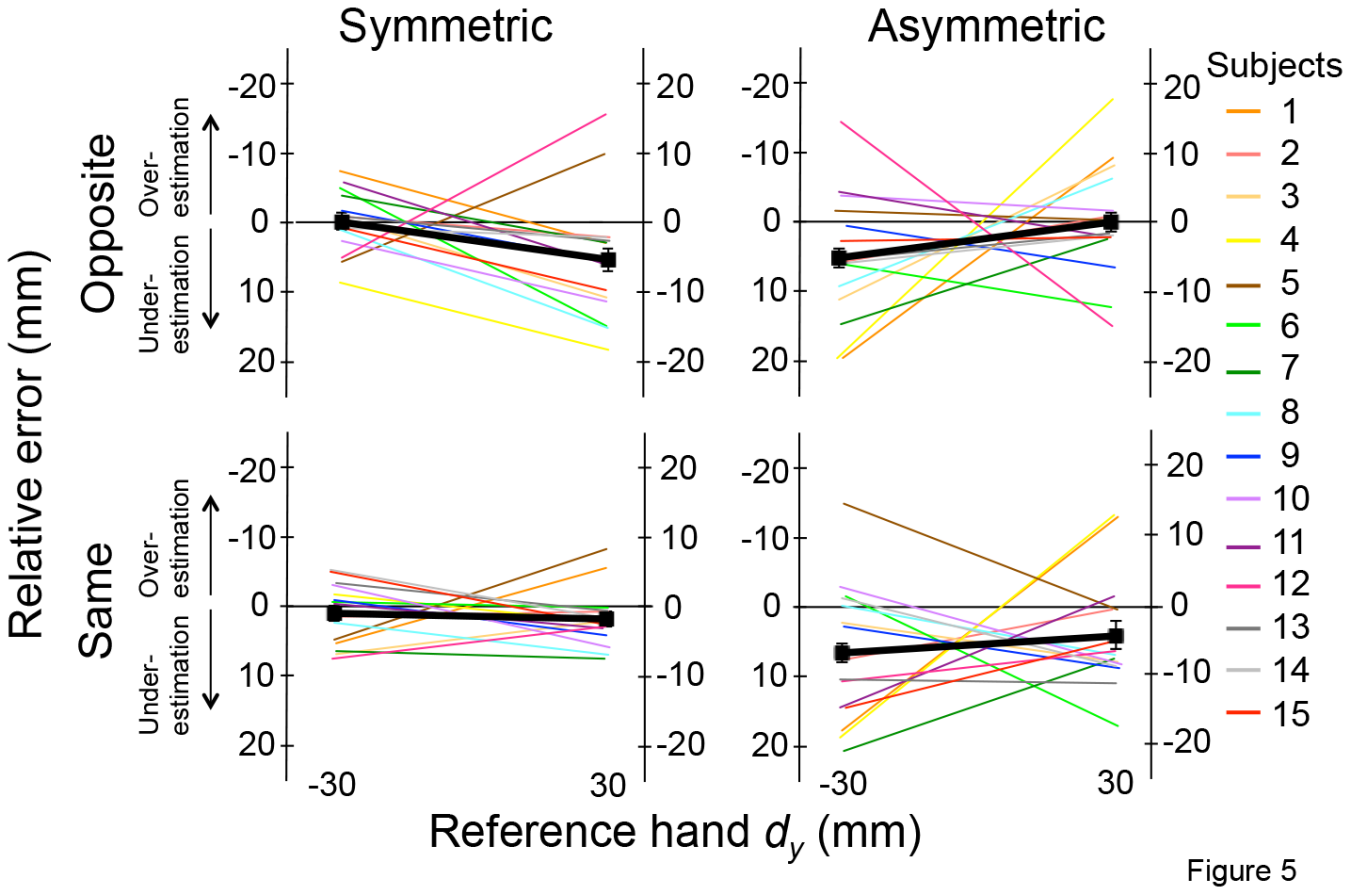
Relative error: individual subjects. Averages of relative errors of 5 trials from each subject are shown as a function of reference hand *d_y_* (−30 and +30 mm) for each of the four matching conditions. The thick black line denotes the mean relative error averaged across 15 subjects with standard error of the mean. Data from the collinear condition were excluded (see text for more details). The left- and right-hand *y*-axes for each plot refer to relative errors obtained for reference hand *d_y_* of −30 and 30 mm, respectively, in which positive or negative relative error are defined as under-estimation errors, respectively.

**Figure 6:**
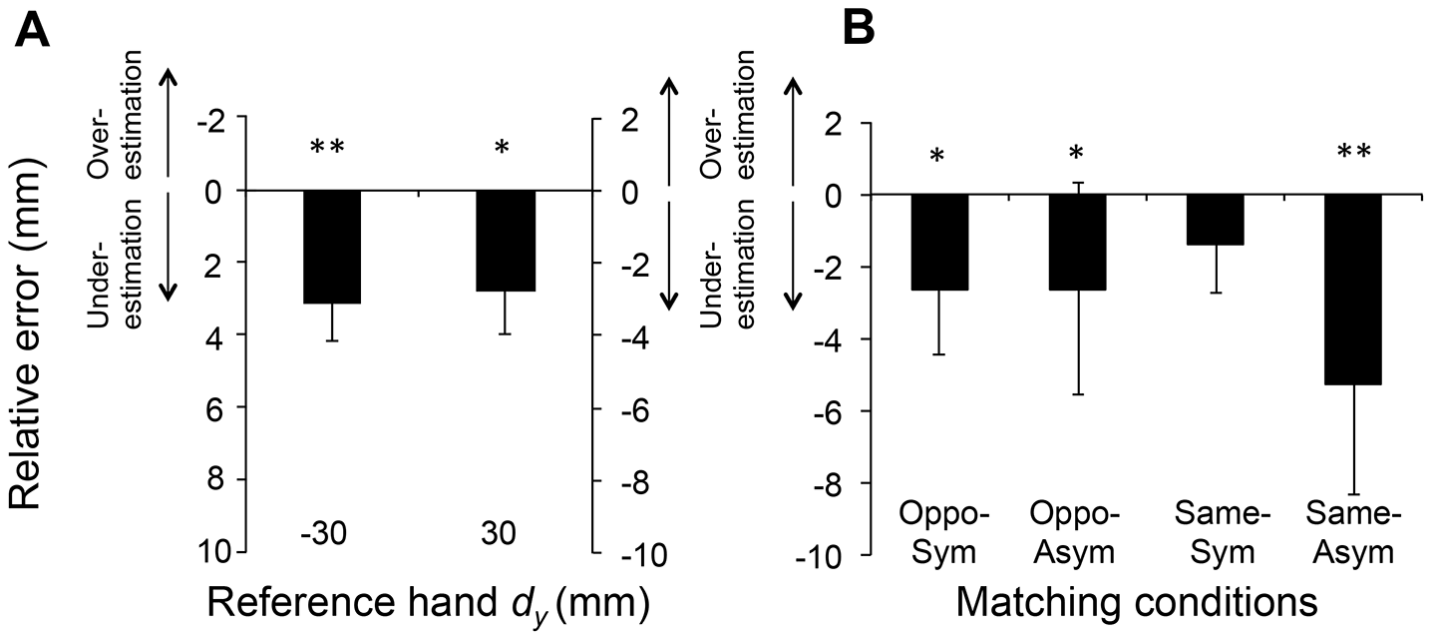
Relative error: averaged data. Relative errors were compared across reference hand *d_y_*s and matching conditions (panels A and B, respectively). For Panel A, the relative errors with respect to the under- and over-estimation are shown in the same format as Figure 5. For Panel B, relative error values were pooled across non-collinear *d_y_*. For both panels, data are averages of all subjects within a given group (±SE). Note that relative error from the collinear condition was excluded from statistical analysis across matching conditions (see text for more details). Single and double asterisks denote a statistically significant difference from zero (*p*<0.05 and 0.01, respectively). Note that, since the opposite signs of relative error were defined as under- and over-estimation, the sign of relative error when reference hand thumb was placed lower (*d_y_* = −30 mm) is inverted for the relative error pooled across the four conditions (Panel B) for graphical purpose only such that the negative relative error always denotes underestimation.

Figure 5 shows the averages of relative matching error of 5 trials from all subjects as a function of *d_y_* without the collinear digit position for each experimental condition. Similar to Figure 3, each line denotes one subject, and the thick black line denotes the mean relative error averaged across 15 subjects. Overall, under-estimation relative error occurred when reference hand thumb was placed higher or lower than the index finger (*d_y_* = 30 or −30 mm), respectively, in all four conditions. Note that the relative error in the collinear condition was excluded from the analysis of directional bias (see Methods). For all but the Same-Symmetric condition, two-tailed *t*-tests revealed under-estimation relative error that was significantly different from zero (*d_y_* = −30 mm: 3.1±1.0 mm; t[_14_] = −3.081; *p*<0.01; *d_y_* = 30 mm: −2.8±1.1 mm; t[_14_] = −2.457, *p*<0.05; Fig. 6A) and in the three matching conditions (Opposite-Symmetric: t[_14_] = −2.146; *p*<0.05, Opposite-Asymmetric: t[_14_] = −3.098; *p*<0.01, Same-Asymmetric: t[_14_] = −4.234; *p*<0.01; Fig 6B). Thus, these findings indicate that subjects tended to underestimate reference hand *d_y_* in all conditions with the exception of Same-Symmetric condition.

## Discussion

The main findings of this study, summarized in Table 1, are that errors in haptic-motor transformations of finger pad distance are sensitive to *(1)* the congruence between the posture of the hand used for sensing and that used for reproducing finger pad distance (greater error for inverse than congruent postures), *(2)* whether finger pad distance is reproduced with the same hand used for sensing (greater error for matching performed with the opposite than same hand), and *(3)* the relative position of contacts (greater errors for collinear than non-collinear finger pad positions). We discuss these results in the context of the role of digit placement sensing for force modulation required for dexterous manipulation.

**Table 1:** Task differences across experimental conditions and rank of matching error.

	Experimental conditions			
	Same-Sym	Oppo-Sym	Same-Asym	Oppo-Asym
Incongruent postures between R*_hand_* and T*_hand_*?	NO	NO	YES	YES
Transfer across hemispheres?	NO	YES	NO	YES
Rank of matching error (smallest to largest)	1	2	3	4

Sym: Symmetric; Asym: Asymmetric: Oppo: Opposite; R*_hand_*: Reference hand; T*_hand_*: Test hand.

### Effect of Hand Posture: Congruent vs. Inverse Hand Configurations (Table 1, Top Row)

The greater absolute error in the asymmetric condition indicates that congruent sensory feedback arising from similar hand postures facilitates the reproduction of sensed *d_y_*. Specifically, when reference and test hand postures were congruent, subjects might have merely tried to duplicate reference hand configuration by matching the remembered feedback rather than perceived *d_y_*, thus bypassing higher-order processing of CoP distance based on sensing CoP of each digit. Therefore, the Asymmetric condition is a more reliable measure of subjects' ability to integrate sensory feedback to estimate *d_y_* regardless of postural sensory cues. It follows that higher-level processing of sensory inputs to estimate finger pad distance leads to greater haptic-motor transformation errors. This conclusion predicts that tasks that require transferring sensory information about digit placement from one hand to another would be performed with greater accuracy when hand postures are mirror symmetric. Examples of such tasks are unimanual tasks where an object is transferred across hands, or bimanual tasks that involve symmetrical application of forces/torques with both hands through similar contact distributions.

### Effect of Hand Used for Sensing and Reproducing Finger Pad Distance: Opposite Versus Same Matching (Table 1, Middle Row)

We found that absolute error was greater in the matching condition using the opposite than same hand. Furthermore, we found that absolute error was smaller in the Symmetric condition using the same hand (Same-Sym) than the other three matching conditions (Fig. 4C). These findings indicate that the perceived sensory information is less accurately transferred across than within hands. This result is consistent with previous studies in which subjects matched wrist [24] and elbow [26]–[28] flexion and extension angles across limbs. However, our task can be considered more complex due to the requirement of integrating the spatial relation between digits' CoP to estimate their vertical distance. It has been suggested that transferring sensory information across hemispheres may increase noise and potential loss of information due to the asymmetry of hemispheric activation during hand movement [23], [29]. This asymmetric activation of hemispheres might have contributed to the greater error found for the Opposite condition, although further work is needed to identify the underlying neural mechanisms.

### Relative Error

Computation of relative error revealed a tendency for underestimating reference hand *d_y_* in most of matching conditions except the Symmetric condition performed with the same hand (Fig. 6). This phenomenon has been observed when the wrist angle of the right hand is matched using the left hand [24]. Despite the task differences (see above), it would appear that transfer of sensory information from the left to the right cerebral hemisphere leads to under-estimation of joint angle, as well as higher-order sensorimotor transformations required when hand postural sensory cues cannot be used to match *d_y_* across hands (Opposite-Asymmetric condition).

Regarding the retrieval of remembered sensory information and matching with the same hand used for sensing *d_y_* (Same condition), there was no directional bias when the matching task was performed *symmetrically* using the same hand, which is also consistent with previous findings on wrist angle matching [24]. However, we also found underestimation in reference hand *d_y_* when the matching task was performed *asymmetrically* using the same hand, which is a condition that cannot be tested in the single joint angle matching task. Thus, we speculate that higher-level processing of finger pad distance based on digit CoP sensing is the primary source of underestimation error when the matching task was performed asymmetrically using the same hand.

### Effect of Relative Digit Position: Collinear vs. Non-collinear Contacts

We found that subjects make greater errors in reproducing finger pad distance (*d_y_*) when sensing collinear than non-collinear contacts. Here we discuss potential neural mechanisms that might underlie these results.

#### Skin Afferents

It is possible that subjects sensed and reproduced non-collinear *d_y_* with greater accuracy due to the greater extent of skin stretch on the dorsal region of the hand. Skin afferent input is likely to play a significant role in sensing digit position in the present experiment as we prevented visual feedback of the hand and ensured consistent deformation of the finger pads by having subject exert similar contact forces across all conditions. Previous studies [20], [22] have shown that the discharge rate of cutaneous receptors, particularly the slowly adapting receptors, increases as a function of skin stretch for the receptors located near the metacarpo-phalangeal (‘*mcp*’) joint of the index finger. Matching performance in our task might have resulted not only from feedback delivered by skin afferents from the dorsal region of the hand, but also on tactile input elicited by deformation of the glabrous skin of the finger pad. However, the contribution of the cutaneous receptors within the contact area to sense CoP should have been largely comparable across all experimental conditions as we controlled contact forces and verified that small trial-to-trial force fluctuations had no influence on matching error. Furthermore, Edin and Johansson (1995) reported that the changes in the skin stretch contributed to an accurate estimation of the static proximal inter-phalangeal joint angle even when tactile feedback provided unreliable information due to anesthesia. Therefore, it is likely that the contribution of skin stretch afferent responses can account for our results, if we assume that our non-collinear *d_y_* elicited a greater discharge from skin afferents, hence a greater afferent signal-to-noise ratio of finger pad position, than collinear *d_y_*.

#### Joint Receptors

Joint receptors of the *mcp* joint of index finger and carpometacarpal (‘*cmc*’) joint of thumb might also have contributed to sensing *d_y_* as they are relatively less active at the mid-range of motion of joint but significantly active towards the limits of the joint range of motion [19], [20], [30], [31]. The joint in the collinear digit position is thought to be at a mid-range of motion of the *mcp* and *cmc* joints, whereas the non-collinear digit positions are closer to the limit of the thumb and index finger *mcp* and *cmc* joint range of motion.

#### Central Commands

In addition to the above-mentioned afferents contribution in sensing the digit position, it has been reported that central motor commands contribute to position sense [32]–[38]. Physiological evidence indicates that central and peripheral signals are strongly correlated due to alpha-gamma co-activation [39]–[41] Furthermore, it has been proposed that predicted future sensory states are implemented through the muscle spindles to update the motor commands during point-to-point movements [42]–[45]. Since we passively positioned the digits and controlled for digit contact forces, the extent to which central commands might have been involved in estimating digit position was likely constant across experimental conditions.

In summary, based on the above arguments we speculate that the smaller error found for non-collinear digit positions might have resulted primarily from the integration of sensory inputs from skin and joint receptors.

### Haptic-motor Transformations: Sensing and Reproducing Finger Pad Distance

To successfully perform our matching task, subjects had to first accurately sense the CoP of each digit of the reference hand, integrate that feedback into an internal representation of distance between CoPs, hold the representation in memory, transfer it to the contralateral cerebral hemisphere (Opposite condition only), and lastly send motor commands to the test hand for controlling the position of each finger pad such as to reproduce the sensed *d_y_*. The errors we report in this process of transforming digit position could have arisen at one or more of these stages, ranging from purely sensing to motor, or at the high-level computation of CoP vertical distance. The fact that Asymmetric and collinear contacts conditions were characterized by greater matching errors suggests sub-optimal transformations at both the high-level computation levels and sensing, respectively. Similarly, tasks involving *d_y_* sensing and reproduction with the same hand might have an advantage as no across-cerebral hemisphere transfer of sensed *d_y_* is required, thus suggesting that retrieval of *d_y_* internal representations is characterized by less noise than retrieving and transferring it to the contralateral hemisphere. However, since our matching task did not involve a significant digit force production when subjects perceived the digit position such as to prevent an object from slipping, further work is needed to address potential contributions of motor commands responsible for digit force production.

### Role of Digit Position Sensing for Dexterous Manipulation

The present study revealed maximum absolute errors of up to ∼1.6 cm (Fig. 4A), and smaller errors for particular combinations of task conditions, e.g., Same-Symmetric (∼0.6 cm). These findings not only provide insight into the capability of the central nervous system (CNS) to use somatosensory feedback for haptic-motor transformation errors, but also about potential mechanisms that the CNS would have to use to ensure successful performance of dexterous manipulation.

For small position sensing errors, the compliance of finger pads might be sufficient to compensate for digit force magnitude and/or direction modulated to the perceived, as opposed to actual, digit contact distribution. However, for greater digit position errors that might occur when contacts of one or more digits are blocked from view by the object (i.e., a scenario similar to our present study), one would expect greater and more detrimental manipulation performance errors. This is because, for a desired set of net forces and torques on the object, the CNS has to compensate for potential trial-to-trial variability in digit position by modulating contact forces accordingly [6]. Conversely, if the CNS only used sensorimotor memories of previously used forces and retrieved them on subsequent manipulations but exerted them at different contacts, object dynamics would differ from that experienced on previous trials. As subjects do modulate forces as a function of variable digit placement [6], [7], the present observations point to the involvement of sensorimotor mechanisms, and these might potentially include vision, capable of compensating for haptic-motor transformation errors. Besides vision of contacts, which does not seem to play a significant role in sensing the orientation of contacts [14], another potential source of feedback that might reduce digit position sensing errors at the onset of manipulation is the intensity and/or pattern of tactile feedback elicited by exerting contact forces.

Trial-to-trial variability in digit placement when grasping was followed by object lifting was smaller than the matching errors found in the present study [6]. This phenomenon may be task-sensitive since there was no requirement to lift the object in the present study. Furthermore, a major difference between previous and present work is that the digits were passively moved by the experimenter at given distances and with no visual feedback of the hand and object. In contrast, in grasp-to-lift tasks subjects are actively changing the vertical distance between the fingertips and are likely to use vision to guide digit placement. When actively modulating fingertip distance, subjects might use a feedforward control strategy whereby a sense of digit placement might already be established before contact with the object (hence, tactile feedback) occurs. We speculate that availability of visual feedback and voluntary modulation of fingertip distance are the main causes underlying the differences in accuracy of digit placement between grasp-and-lift tasks and the present matching task.

In summary, the present errors associated with haptically-based reproduction of finger pad distance indicate that the CNS must implement mechanisms to compensate for errors in sensing finger pad distance to ensure that digit forces are distributed according to the required manipulation task requirements. The extent to which these mechanisms might include vision of the hand and/or tactile feedback is the subject of ongoing investigation.
